# Empagliflozin Effectively Attenuates Olanzapine-Induced Body Weight Gain in Female Wistar Rats

**DOI:** 10.3389/fphar.2021.578716

**Published:** 2021-04-19

**Authors:** Ghulam Md Ashraf, Badrah S. Alghamdi, Fahad S. Alshehri, Mohammad Zubair Alam, Haythum O. Tayeb, Frank I. Tarazi

**Affiliations:** ^1^ Pre-Clinical Research Unit, King Fahd Medical Research Center, King Abdulaziz University, Jeddah, Saudi Arabia; ^2^ Department of Medical Laboratory Technology, Faculty of Applied Medical Sciences, King Abdulaziz University, Jeddah, Saudi Arabia; ^3^ Department of Physiology, Neuroscience Unit, Faculty of Medicine, King Abdulaziz University, Jeddah, Saudi Arabia; ^4^ Department of Pharmacology and Toxicology, College of Pharmacy, Umm Al-Qura University, Makkah, Saudi Arabia; ^5^ Division of Neurology, Department of Internal Medicine, King Abdulaziz University, Jeddah, Saudi Arabia; ^6^ Department of Psychiatry and Neurology, Harvard Medical School and McLean Hospital, Belmont, MA, United States

**Keywords:** antidiabetics, antipsychotics, body weight gain, empagliflozin, olanzapine, wistar rats, gender difference

## Abstract

Atypical antipsychotic drugs are commonly associated with undesirable side effects including body weight gain (BWG) and metabolic deficits. Many pharmacological interventions have been tested in an attempt to minimize or prevent these side effects. Preliminary evidence suggests that antidiabetic drugs may be effective in attenuating antipsychotic-induced BWG. In the current study, we examined the effect of an antidiabetic drug empagliflozin (EMPA) on BWG induced by anatypical antipsychotic drug olanzapine (Ola) in female and male Wistar rats. Rats were divided into six groups based on the dose they received: group 1 (female control), group 2 (female EMPA, 20 mg/kg; IG), group 3 (female Ola, 4 mg/kg; IP), group 4 (female Ola, 4 mg/kg; IP + EMPA, 20 mg/kg; IG), group 5 (male control), and group 6 (male Ola, 4 mg/kg; IP). Ola induced sustained increase in BWG. The subsequent treatment of Group 3 and 4 with EMPA attenuated the Ola-induced BWG in female Wistar rats. In terms of the gender difference between female and male Wistar rats, the male control group 5 gained more weight throughout the study as compared to the female control group 1. Similarly, the male Ola group 6 gained more weight throughout the study as compared to the female Ola group 3. However, Ola did not cause any weight difference between male rats treated with Ola in comparison with male control group, thus showing a significant gender difference regarding body weight between male and female Wistar rats regardless of Ola administration. In addition, the present findings showed that EMPA effectively attenuates the Ola induced BWG in female Wistar rats. These novel findings should help to better understand the underlying molecular and behavioral mechanisms contributing to the observed increase in body weight after treatment with Ola and other atypical antipsychotic drugs across male and female rats.

## Introduction

Atypical antipsychotics are the first line of pharmacotherapy for patients with schizophrenia and other idiopathic psychotic disorders (Geddes et al., 2000). Therefore, long-term administration of antipsychotics such as Olanzapine (Ola) risperidone and other agents is necessary to ensure proper management of symptoms (Junghan et al., 1993; Duggan et al., 2005). However, prolonged administration of antipsychotics has been associated with body weight gain (BWG) (Liu et al., 2017a). Antipsychotic-induced BWG can lead to patients’ non-compliance and failure to continue antipsychotic drug treatment. Indeed, patient non-compliance to treatment is a significant challenge for clinicians treating schizophrenia (Weiden and Olfson, 1995). The metabolic deficits caused by antipsychotics increase the risk of cardiovascular and cerebrovascular related conditions (Dayabandara et al., 2017; Barton et al., 2020). This is a critical matter as studies have shown that cardiovascular diseases are the leading cause of death in patients with schizophrenia (Correll et al., 2017; Hayes et al., 2017; Westman et al., 2018). There is no clear relationship between BWG and an antipsychotic dose; however, agents which increase BWG appear to increase appetite, which leads to a signficant increase in calorie intake and eventually body weight (Werneke et al., 2013; Tungaraza, 2016).

Ola is a thienobenzodiazepine derivate that is effective managing the symptoms of schizophrenia and reducing the psychopathological symptoms of psychosis. It is also effective in controlling the acute manic episodes associated with bipolar disorder, and have provided some therapeutic advantages over other antipsychotic agents (Citrome et al., 2019). However, Ola administration has been reported to induce profound BWG accompanied with higher incidence of metabolic deficits, such as hypertension, diabetes and hyperlipidemia, as compared to other antipsychotic agents (Mauri et al., 2014). Adjunctive treatment with other agents that can minimize or normalize Ola-induced BWG can enhance the safety and tolerability profiles of an effective antipsychotic, thus highlighting the need to develop improved therapies or interventions to minimize these side effects.

Earlier studies suggested that antidiabetic drugs can mitigate glucose intolerance caused by Ola in female rats which have been consistent with those reported in preclinical and clinical studies (Boyda et al., 2012). Many studies investigating the effect of antidiabetic drugs on Ola induced physiological changes suggested the need for further studies on the effect of new antidiabetic drug combinations in patients treated with antipsychotics (Boyda et al., 2014a). The same group showed that routine exercise reduces Ola-induced glucose intolerance and increases skeletal muscle levels of GLUT 4, the insulin-responsive transporter that mediates glucose uptake into cells (Boyda et al., 2014b). Therefore, antidiabetic drugs have been clinically suggested to attenuate the metabolic side effects of antipsychotics and especially BWG (Dayabandara et al., 2017). A meta-analysis of 12 published studies found that antidiabetic drugs such as metformin improved metabolic parameters in patients treated with antipsychotics (de Silva et al., 2016). These studies encouraged the evaluation of other antidiabetic agents as adjunctive therapies to minimize Ola-induced BWG. Therefore, we investigated the effect of Empagliflozin (EMPA) in attenuating the Ola induced BWG in both male and female Wistar rats. EMPA is the third-generation antidiabetic drug acting as sodium-glucose transport protein two inhibitor (SGLT2), which provides a new mechanism of action to improve glycemic control with modest decreases in systolic blood pressure and body weight (Baar et al., 2018; Pradhan et al., 2019). The effects of EMPA on Ola-induced BWG have not been determined and require further investigation. In an earlier study, we standardized the effective correlated dosage of Ola (4 mg/kg/OD) and EMPA (20 mg/kg/OD) in female Wistar rats (Ashraf et al., 2020). In the present study, we employed these standardized concentrations to investigate the effect of EMPA on Ola-induced BWG in female and male Wistar rats. We hypothesized that EMPA will effectively attenuate Ola-induced BWG in female Wistar rats.

## Materials and Methods

### Animal Subjects

A total of 60 (40 female and 20 male) Wistar rats, weighing approximately 190 g (∼3 months old) were procured from the animal house of King Fahad Medical Research Center (KFMRC), King Abdulaziz University (KAU), Jeddah, Saudi Arabia. Rats were divided into six groups of rats (*n* = 10) each. The animals were kept in standard laboratory conditions of 12 h light/dark cycle (7 am and 7 pm), controlled humidity (30–70% relative humidity) and temperature (18–26°C). The animals had free access to water and chow (normal standard rodent chow) under well supervised and inspected animal house facility at KFMRC. All the experiments were reviewed and approved by the Unit of Biomedical Ethics Research Committee (Reference No. 389–19), Faculty of Medicine, KAU, Jeddah, Saudi Arabia.

### Drugs and Procedures

The drugs Ola (Zyprexa, Lilly) and EMPA (Jardiance, Lilly) were obtained from Nahdi pharmacy in Jeddah. Ola was given at a dose of 4 mg/kg; intraperitoneal (IP), once daily (OD). EMPA was given at a dose of 20 mg/kg; intragastrical (IG) by oral gavage needle OD in 0.5% aqueous hydroxyethyl cellulose (HC). These doses were chosen based on previous study done in our lab (Ashraf et al., 2020). The Ola was prepared by dissolving crushed tablets according to the methodology described in a previous study (Kurbanov et al., 2012). The active component of Ola was measured in each tablet and then tablets were crushed. A buffer solution of 0.1 M sodium hydroxide and distilled water was prepared fresh every 2 days, the pH was adjusted to 5.5 and kept refrigerated at 5°C. Fresh drug preparation was made every day by dissolving the required amount of crushed Ola tablets in the buffer solution to reach the injection volume of 2 ml/kg/rat. Before injections, the drug solution was vortexed for resuspension. For control groups (1 and 5), only buffer solution was given in a volume of 2 ml/kg/rat. The treatment schedule has been summarized in Table 1.

**TABLE 1:** Treatment schedule for all male and female groups of the experiment.

	Female group				Male group	
	Group 1	Group 2	Group 3	Group 4	Group 5	Group 6
	Control	EMPA	Ola	Ola + EMPA	Control	Ola
	10 rats	10 rats	10 rats	10 rats	10 rats	10 rats
Week 1–2	Buffer (IP)	Buffer (IP)	Ola (4 mg/kg; IP) in buffer	Ola (4 mg/kg; IP) in buffer	IP buffer	Ola (4 mg/kg; IP) in buffer
Week 3–4	Buffer (IP) + HC (IG)	Buffer (IP) + EMPA (20 mg/kg; IG) in HC	Ola (4 mg/kg; IP) in buffer +Cellulose (IG)	Ola (4 mg/kg; IP) in buffer + EMPA (20 mg/kg; IG) in HC	IP buffer	Ola (4 mg/kg; IP) in buffer

EMPA, empagliflozin; HC, hydroxyethyl cellulose; IP, intraperitoneally; IG, intragastrically; Ola, olanzapine.

### Groups

o Group 1 (Female control) was injected daily at 1:00 pm with the IP vehicle from day 1–15. From day 16–28, with the continuation of vehicle IP injections, all rats were administered orally with 0.5% aqueous HC at 2:00 pm daily.
o Group 2 (Female EMPA) was injected daily at 1:00 pm with the IP vehicle from day 1–15. From day 16–28, with the continuation of vehicle IP injections, all rats were given EMPA (20 mg/kg; IG) with 0.5% aqueous HC at 2:00 pm daily.
o Group 3 (Female Ola) was injected daily at 1:00 pm with the Ola (4 mg/kg; IP) from day 1–15. On day 16, with the continuation of Ola regimen, rats were administered orally with 0.5% aqueous HC at 2:00 pm daily.
o Group 4 (Female Ola + EMPA) was injected daily at 1:00 pm with the Ola (4 mg/kg; IP) from day 1–15. On day 16, with the continuation of Ola regimen, rats were given EMPA (20 mg/kg; IG) in HC at 2:00 pm daily.
o Group 5 (Male control) was injected daily at 1:00 pm with the IP vehicle from day 1–28.
o Group 6 (Male Ola) was injected daily at 1:00 pm with the Ola (4 mg/kg; IP) from day 1–28.

### Daily and Weekly Measurements

Food consumption and water intake were measured daily per cage. Any remnant of food bites on the floor of the cage was collected and measured as well. Weight was measured daily at 08:00 am. Plasma samples were collected weekly.

### Animal Sacrifice

The rats were sacrificed on 28th day. Brain, liver and fecal samples were collected and stored in −80°C for future studies.

### Statistical Analysis

BWG was calculated as:

$$\%\;\mathrm{BWG}\;\mathrm{from}\;\mathrm{day}\;\text{'}0\text{'}=\frac{\mathrm{new}\;\mathrm{weight}-\mathrm{weight}\;\mathrm{at}\;\mathrm{day}\;\text{'}0\text{'}}{\mathrm{weight}\;\mathrm{at}\;\mathrm{day}\;\text{'}0\text{'}}\mathrm{\times 100}.$$

All the data were analyzed using two-way repeated-measures ANOVA (days × treatment). Tukey’s test was used post hoc to identify significant changes in the percentage of BWG, water and food consumption in female rats as well as gender difference. Tukey post hoc test was used to identify significant changes in the percentage of BWG, water and food consumption in male rats. A *p*-value of less than 0.05 was set for significance. All data were analyzed using GraphPad Prism 8.4.3.

## Results

### Effect of EMPA on Ola-Induced BWG in Female Wistar Rats

**FIGURE 1:**
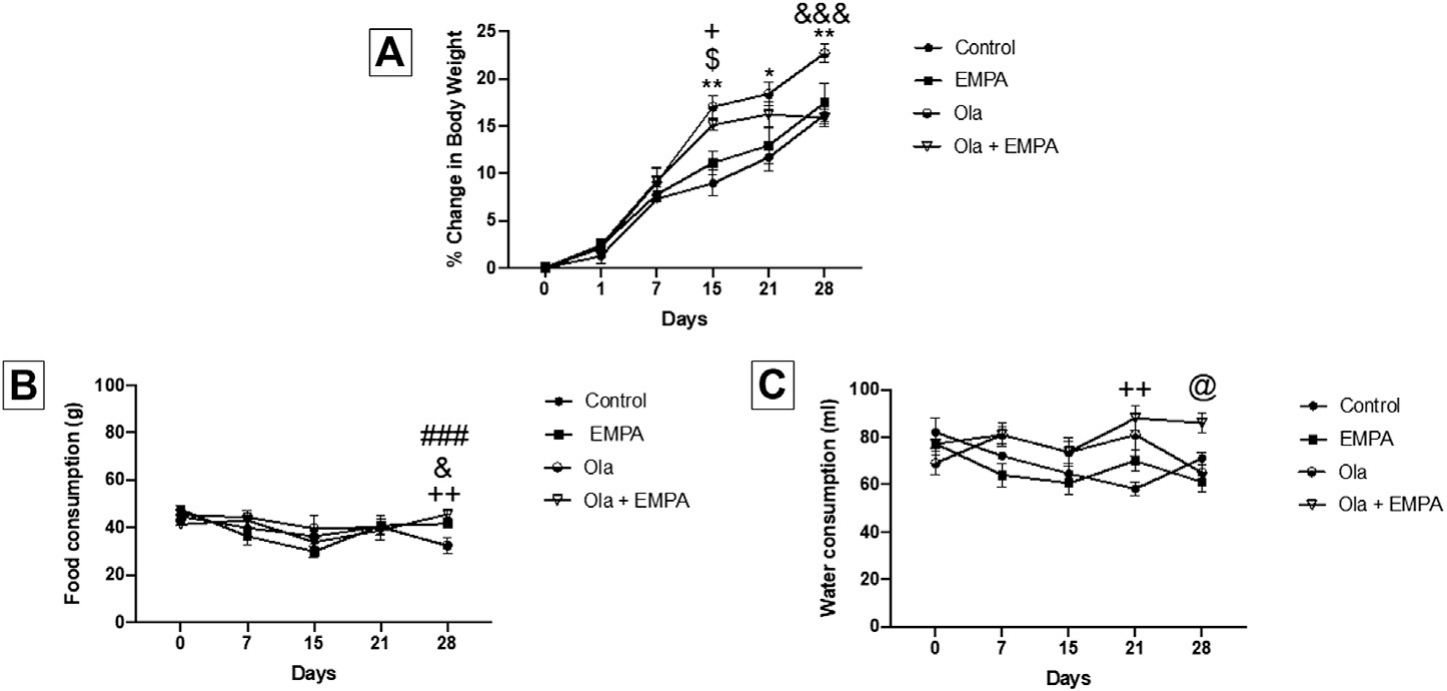
**(A)** The effect of Ola on the percentage of change in body weight on female rats as compared to the other groups. * represent the significant difference between control and Ola group at *p* > 0.05; $ represent the significant difference between EMPA and Ola group at *p* > 0.05; + represent the significant difference between control and Ola + EMPA group at *p* > 0.05; and represent the significant difference between Ola and Ola + EMPA group at *p* > 0.05 **(B)** The effect of Ola on the food consumption on female rats as compared to the other groups. + represent the significant difference between control and Ola + EMPA group at *p* > 0.05; and represent the significant difference between Ola and Ola + EMPA group at *p* > 0.05; # represent the significant difference between control and EMPA group at *p* > 0.05 **(C)** The effect of Ola on the water consumption on female rats as compared to the other groups. + represent the significant difference between control and Ola + EMPA group at *p* > 0.05; @ represent the significant difference between EMPA and Ola + EMPA group at *p* > 0.05.

The effect of Ola on female Wistar rats was measured as the percent change in body weight (Figure 1A). Two-way RM ANOVA showed a significant effect of days × treatment [F (15, 135) = 4.260, *p* < 0.0001], days [F (2.728, 73.66) = 266.1, *p* < 0.0001], and treatment [F (3, 27) = 6.001, *p* = 0.0029]. Further analysis using the Tukey *post hoc* test revealed that Ola induced sustained increase in BWG in group 3 (female Ola) as compared to group 1 (female control) at day 15 (*p* = 0.0038), day 21 (*p* = 0.0166), and day 28 (*p* = 0.0020). Similarly, Ola induced sustained increase in BWG in group 4 (female Ola-4 + EMPA-20) compared to the group 1 (female control) at day 15 (*p* = 0.0110) before starting the treatment with EMPA. Interestingly, EMPA treatment was able to attenuate the Ola-induced BWG, as observed in group 4 (female Ola-4 + EMPA-20) compared with the group 3 (female Ola-4) (*p* = 0.0007). On the other hand, no changes were observed in group 2 (female EMPA-20) as compared to group 1 (female control) throughout the study.

### Effect of EMPA and Ola on Food Consumption in Female Wistar Rats

The effect of Ola and EMPA on food consumption in female Wistar rats was measured as the amount of food consumed during the 24 h (Figure 1B). Two-way RM ANOVA showed a significant effect of days × treatment [F (12, 56) = 2.432, *p* = 0.0127], and days [F (2.897, 40.56) = 6.667, *p* = 0.0010]; however, it showed no effect of only treatment [F (3, 14) = 0.2767, *p* = 0.8412]. Further analysis using the Tukey post hoc test showed that EMPA-20 caused an increase in food consumption in group 2 (female EMPA) as compared to group 1 (female control) at day 28 (*p* = 0.0006); however, no changes were observed on other days. Besides, the combination of Ola-4 and EMPA-20 caused an increase in the food consumption in group 4 (female Ola-4 + EMPA-20) as compared to group 1 (female control) at day 28 (*p* = 0.0045); however, no changes were observed on other days. Likewise, the combination of Ola and EMPA caused an increase in food consumption in group 4 (female Ola-4+ EMPA-20) compared to the group 3 (female Ola-4) at day 28 (*p* = 0.0397); however, no changes were observed on other days.

### Effect of EMPA and Ola on Water Intake in Female Wistar Rats

The effect of Ola and EMPA on water intake in female Wistar rats was measured as the amount of water consumed during the 24 h (Figure 1C). Two-way RM ANOVA showed a significant effect of days × treatment [F (12, 64) = 2.852, *p* = 0.0034], treatment [F (3, 16) = 6.471, *p* = 0.0045]; however, no effect of number of days [F (3.037, 48.59) = 2.179, *p* = 0.1018]. Further analysis using the Tukey *post hoc* test showed an increase in water consumption in group 4 (female Ola-4 + EMPA-20) as compared to group 1 (female control) at day 21 (*p* = 0.0082) and compared to group 2 (female EMPA-20) at day 28 (*p* = 0.0120).

### Effect of Ola on Weight Gain in Male Wistar Rats

**FIGURE 2:**
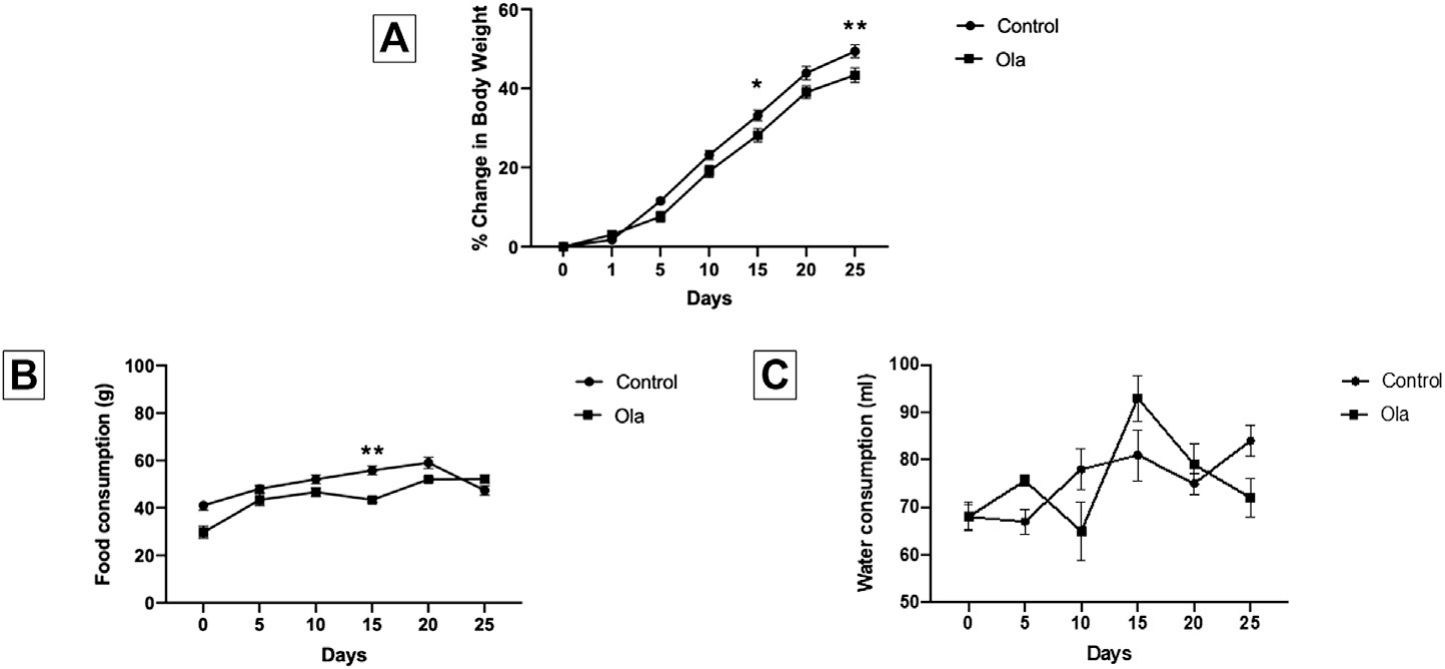
**(A)** The effect of Ola on the percentage of change in body weight on male rats as compared to the control group **(B)** The effect of Ola on the food consumption on male rats as compared to the to the control group **(C)** The effect of Ola on the water consumption on male rats as compared to the control group. * represent the significant difference between control and Ola group at *p* > 0.05.

The effect of Ola on weight of male Wistar rats was measured as the percent change in body weight (Figure 2A). Two-way RM ANOVA showed a significant effect of days × treatment [F (6, 108) = 5.798, *p* < 0.0001], days [F (6, 108) = 1,045, *p* < 0.0001], and treatment [F (1, 18) = 4.845, *p* = 0.0410]. Further analysis using the Sidak *post hoc* test revealed that Ola-4 did not cause any weight gain; however, induced reduction on weight gain at day 15 (*p* = 0.0464) and day 25 (*p* = 0.0081).

### Effect of Ola on Food Consumption and Water Intake in Male Wistar Rats

The effect of Ola on food consumption and water intake in male Wistar rats was measured as the amount of food and water consumed during the 24 h. Two-way RM ANOVA on food consumption showed a significant effect of days × treatment [F (5, 40) = 5.902, *p* = 0.0004], days [F (2.835, 22.68) = 28.46, *p* < 0.0001], and treatment [F (1, 8) = 16.93, *p* = 0.0034]. Further analysis using the Sidak *post hoc* test revealed that Ola-4 caused a decrease in food consumption only on day 15 (*p* = 0.0059) (Figure 2B). On the other hand, Two-way RM ANOVA on water intake showed a significant effect of days × treatment [F (5, 40) = 3.891, *p* = 0.0057], and days [F (3.333, 26.67) = 6.563, *p* = 0.0014]; however, no effect on treatment [F (1, 8) = 0.0006232, *p* = 0.9807] (Figure 2C).

### Effect of Gender Difference on Ola Induced Weight Change in Male and Female Wistar Rats

**FIGURE 3:**
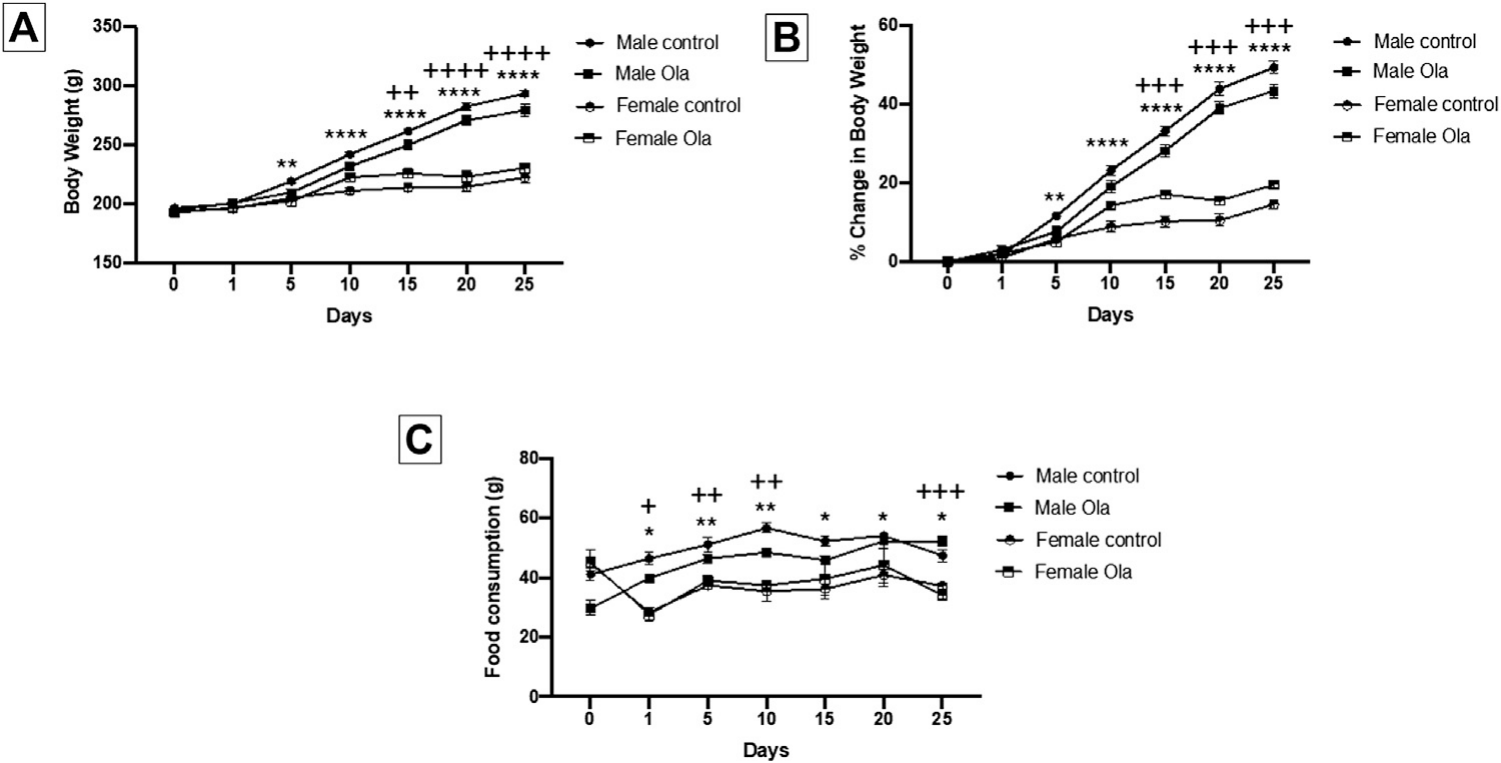
**(A)** The effect of Ola on the average body weight on male as compared to the female rats **(B)** The effect of Ola on the percentage of change in body weight on male as compared to the female rats **(C)** The effect of Ola on food consumption in male compared to the female rats. * represent the significant difference between the control male and the control female group at *p* > 0.05; + represent the significant difference between the Ola male and the Ola female group at *p* > 0.05.

The effect of Ola on weight of female and male Wistar rats was measured as the percent change in body weight. Two-way RM ANOVA showed a significant effect of days × treatment [F (18, 210) = 72.89, *p* < 0.0001], days [F (2.596, 90.85) = 828.6, *p* < 0.0001], and treatment [F (3, 35) = 79.76, *p* < 0.0001]. Further analysis using the Tukey *post hoc* test revealed an interesting finding between females and males regarding Ola-4 effect on the weight change and percentage of weight change. Group 6 (male Ola-4) gained more weight through the study in comparison to group 3 (female Ola-4) starting from day 5 to day 25 (Figures 3A,B). Similarly, group 5 (male control) gained more weight through the study in comparison to group 1 (female control) starting from day 5 to day 25 (Figures 3A,B). It is important to know that the initial weight for both female and male rats was statistically different across all the tested groups. The effect of Ola on female and male rats’ food consumption was measured as the amount of food consumed in (g). Two-way RM ANOVA showed a significant effect between female and male groups, days × treatment [F (18, 96) = 4.280, *p* < 0.0001], days [F (2.560, 40.96) = 8.042, *p* = 0.0005], and treatment [F (3, 16) = 45.45, *p* < 0.0001]. Further analysis using the Tukey post hoc test, to determine which days were significant, between females and males showed that female rats consumed less food compared to male rats consistently from the beginning of the study until the end of the experiment as shown in Figure 3C.

## Discussion

Atypical antipsychotics are commonly associated with undesirable side effects including BWG and metabolic deficits. Excessive BWG has been reported in approximately 50% of patients treated with dissimilar antipsychotics (Baptista, 2007). Many pharmacological interventions have been tested in an attempt to minimize or prevent these side effects. Preliminary clinical evidence suggests that antidiabetic drugs may be useful in attenuating/reversing the metabolic side effects associated with antipsychotics, especially BWG (Dayabandara et al., 2017). Metformin is being prescribed to improve the disturbed metabolic parameters in patients treated with antipsychotics (de Silva et al., 2016). However, metformin administration has shown serious gastrointestinal side effects resulting in discontinuation of the drug in approximately 5% patients (Rena et al., 2017; Siavash et al., 2017). More recently, FDA alerted clinicians and patients regarding the voluntary recalls of some extended-release (ER) dosage form of metformin drugs because of the presence of a cancer-causing agent N-nitrosodimethylamine (NDMA) above the acceptable intake limit (FDA, 2020; Yang et al., 2020). This encouraged the evaluation of other antidiabetic agents as adjunctive therapies to minimize antipsychotic-induced BWG. EMPA being a relatively newer antidiabetic drug with no report of gastrointestinal side effects, could provide a healthy replacement for metformin, especially for patients suffering from gastrointestinal disorders.

The present study was designed to investigate the effect of EMPA-20 in Ola-4 induced BWG in male and female Wistar rats. Figure 1A depicts Ola-4 induced sustained increase in BWG in group 3 (female Ola-4) and group 4 (female Ola-4 + EMPA-20) as compared to group 1 (female control). These findings confirm that EMPA-20 effectively attenuates Ola-4 induced weight gain in female Wistar rats. The mechanisms behind antipsychotic-induced BWG in female Wistar rats are not yet fully understood and warrants further investigation. Understanding the mechanisms of BWG facilitates aiming specific key targets in finding the best approach to prevent it. Various studies have used several compounds for their potential effects, such antioxidants (Raskind et al., 2007), antidiabetics drugs (Lykkegaard et al., 2008; Hu et al., 2014), antihistamine drugs (Atmaca et al., 2003), antivertigo drugs (Lian et al., 2014), and herbal compounds (Razavi et al., 2017). In this context, our finding of EMPA-20 being an effective attenuator of Ola-induced BWG provides a strong alternative to clinicians who have been co-administering other drugs/agents with limited success.

A noticeable finding in our study was that Ola-4 did not cause any BWG in male Wistar rats (Figure 2A). Figures 2B,C depicts that Ola-4 caused a marginal effect in food consumption and water intake, respectively. Interestingly, group 6 (male Ola-4) gained more weight through the study in comparison to group 3 (female Ola-4) starting from day 5 to day 25 (Figures 3A,B). Similarly, group 5 (male control) gained more weight through the study in comparison to group 1 (female control) starting from day 5 to day 25 (Figures 3A,B). These findings are in agreement with the fact that most of the animal studies were conducted using female rats (Cooper et al., 2005; Kalinichev et al., 2005; Weston-Green et al., 2010; Sejima et al., 2011; He et al., 2014), as male rats did not show any BWG in most cases after long-term Ola treatment (Choi et al., 2007; Cooper et al., 2007). This could be due to different factors between males and females including hormonal differences (Cooper et al., 2005) and other genetic factors which may induce a reduction in locomotor activity (Raskind et al., 2007), hypothalamic changes in controlling food intake (Fernø et al., 2011), induction of insulin resistance (Park et al., 2010), and changes in histamine receptors activity (Kim et al., 2007).

Some studies suggest that SGLT2 inhibitors increase the risk of genital infections, but the effect differs among SGLT2 inhibitors and trials with varying follow up. The meta-analyses of RCTs showed no significant difference in UTIs between SGLT2 inhibitors vs control, but suggested enhanced risk of genital infections with SGLT2 inhibitors (Liu et al., 2017b). However, data from three major outcome trials reported in New England Journal of Medicine clearly suggest that SGLT2 inhibitors are not associated with an increased risk of UTIs (Zinman et al., 2015; Neal et al., 2017; Wiviott et al., 2019). Another recent study reported similar risk of severe and non-severe UTI events among those initiating SGLT2 inhibitor therapy and among patients initiating treatment with a different second line antidiabetic medication (Dave et al., 2019). The analysis of a pooled safety data based on more than 15,000 patient-years’ exposure supports a favorable benefit-risk profile of EMPA in T2DM patients, with more frequent genital infections (but not UTIs) observed in participants treated with EMPA as compared to controls (Kohler et al., 2017). In our study, we did not observe any marked symptoms of genital infection or UTIs, which needs to be investigated more closely in further studies. Moreover, there was zero mortality of rats with no bloody diarrhea, thus suggesting EMPA to be safe from gastrointestinal disorders reported in patients on metformin therapy.

## Conclusion

The antidiabetic drug, empagliflozin, was unexplored for its potential role in attenuating olanzapine-induced BWG in Wistar rats. In this study, we for the first-time report that EMPA is an effective attenuator of Ola-induced BWG in female Wistar rats. The findings of this study will open up new avenues for clinicians administering EMPA is an effective and healthy replacement of metformin and other drugs with some serious side effect in patients treated with atypical antipsychotics like Ola. This, in turn, will boost the underlying molecular and behavioral mechanisms that contribute to antidiabetic drug mediated attenuation of antipsychotic-induced BWG, and can guide the development of novel therapies with superior efficacy, safety and tolerability compared to existing pharmacotherapies.

## Data Availability

The raw data supporting the conclusions of this article will be made available by the authors, without undue reservation.
